# From genomes to interventions: computational strategies transforming parasitology

**DOI:** 10.3389/fvets.2026.1823640

**Published:** 2026-06-19

**Authors:** Khali Mohammed, Asmaa El-kady, Hatem Elshabrawy, Marawan Khodary

**Affiliations:** 1 Department of Epidemiology and Medical Statistics, Faculty of Public Health and Health Informatics, Umm Al-Qura University, Mecca, Saudi Arabia; 2 Department of Medical Parasitology, Faculty of Medicine, Qena University, Qena, Egypt; 3 Department of Molecular and Cellular Biology, College of Osteopathic Medicine, Sam Houston State University, Conroe, TX, United States; 4 Faculty of Medicine, Qena University, Qena, Egypt

**Keywords:** computational genomics, drug discovery, immunoinformatics, *in silico*, machine learning, parasitology

## Abstract

*In silico* techniques have become essential in contemporary parasitology, offering swift, cost-effective, and scalable methods for exploring parasite biology, host–parasite interactions, drug resistance, the discovery of diagnostic markers, vaccine development, and the prioritization of therapeutic targets. Computational frameworks that encompass genomics, pangenomics, phylogenetics, transcriptomics, proteomics, structural biology, molecular docking, molecular dynamics simulations, artificial intelligence (AI), and immunoinformatics have collectively revolutionized parasite research. They enable the systematic identification of conserved antigens, lineage-specific virulence factors, metabolic weaknesses, and potential therapeutics across the primary parasitic disease categories discussed in this review. Simultaneously, the growth of databases and analytical platforms focused on parasites has enhanced genome annotation, inhibitor discovery, epitope prediction, and systems-level analysis. However, despite these advancements, numerous workflows rely on incomplete or inadequately annotated datasets and biologically oversimplified assumptions that fail to accurately represent the complexity of parasites, variations in their life cycles, and host-dependent factors—further complicated by inconsistent data quality and diminished reproducibility across species. As a result, computational findings necessitate thorough integration with transcriptomic, proteomic, structural, and functional experimental data. When utilized in this collaborative manner, *in silico* methods expedite hypothesis generation, refine experimental parameters, and bolster rational approaches for drug discovery, vaccine design, and epidemiological surveillance.

## Graphical abstract

**Figure:**
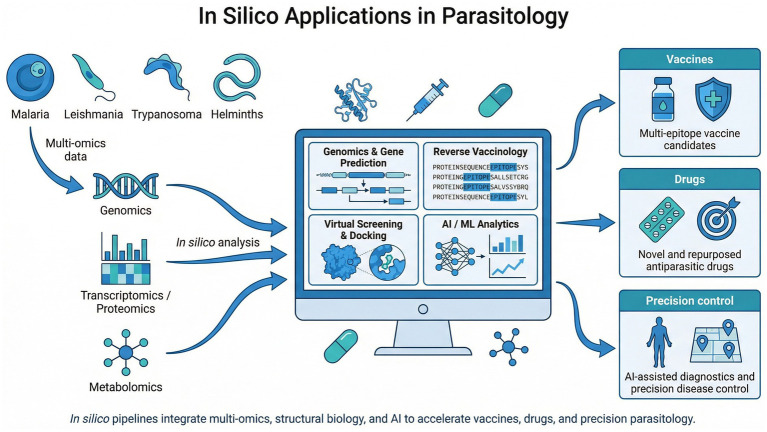

## Highlights

- *In silico* applications are transforming parasitology, spanning fundamental biology through translational research.
- Gene prediction, comparative genomics, and genome-scale metabolic models form the computational backbone for target and pathway discovery.
- Reverse vaccinology pipelines enable rational design of multi-epitope vaccine candidates against major parasitic infections.
- Virtual screening, molecular docking, molecular dynamics, and ADMET prediction collectively accelerate antiparasitic drug discovery and repurposing.
- Systems biology and host–parasite interaction modeling identify synthetic lethal targets and metabolic chokepoints inaccessible to traditional approaches.
- AI and ML are redefining diagnostics, drug resistance surveillance, and epidemiological forecasting in parasite-endemic settings.
- Current methodological limitations and future directions toward precision parasitology—including strain- and patient-specific workflows—are critically evaluated

## 1 Introduction

Parasitic diseases pose a significant global health threat, affecting billions of individuals and leading to considerable morbidity and mortality, especially in developing countries (1). Conditions such as malaria, leishmaniasis, schistosomiasis, and toxoplasmosis continue to impose a substantial socioeconomic burden, worsened by the rise of drug resistance and the absence of effective vaccines for many of the most common infections (2). The conventional approach to antiparasitic research, which heavily depends on resource-demanding *in vivo* and *in vitro* experimental models, has found it challenging to keep up with the evolutionary adaptability of these intricate eukaryotic pathogens (3). This situation has prompted a necessary transformation in research strategy, leading to the adoption of *in silico*methods (4, 5).

The term “*in silico*,” which originates from the Latin term “in silicon,” pertains to research that is conducted entirely through computer means or via computer simulations (6). This field is expanding swiftly and encompasses the global advancement of methods for utilizing software to gather, analyze, and integrate biological and medical data from a variety of sources. More precisely, it delineates the application of this information in the formulation of computational models or simulations that can facilitate predictions, propose hypotheses, and ultimately lead to discoveries or progress in medicine and therapeutics (7). Frequently, the term is also used to indicate the translational use of modelling and simulation, i.e., ‘the direct use of computer simulation in the diagnosis, treatment, or prevention of a disease (8).

In the field of parasitology, the utilization of *in silico* techniques, spanning bioinformatics, computational biology, and artificial intelligence (AI), has transformed the discipline by offering high-throughput, cost-efficient, and predictive tools that facilitate the analysis of parasite biology, the identification of new drug targets, and the acceleration of vaccine development (9, 10). The surge of genomic, transcriptomic, and proteomic data from diverse parasite species, propelled by next-generation sequencing technologies, has established a data-rich landscape where computational analysis is not just an auxiliary component but a critical necessity for deriving biological insights (9, 11, 12).

The shift towards a computational “dry-lab” methodology presents numerous significant benefits. It facilitates the swift evaluation of extensive chemical libraries, the forecasting of protein structures and functions, the simulation of intricate host–parasite dynamics, and the discovery of possible vaccine candidates directly from genomic information (13, 14). This narrative review intends to deliver a thorough overview of the diverse contributions of *in silico* research to contemporary parasitology. We will examine the crucial functions of computational genomics, immunoinformatics, drug development, and sophisticated machine learning strategies, demonstrating how these digital instruments are fundamentally transforming the battle against parasitic illnesses. The following sections will elaborate on these applications, emphasizing essential methodologies, significant milestones, and the obstacles that need to be overcome to fully harness the potential of the digital landscape in parasitology.

## 2 Scope and search strategy

A comprehensive literature search was performed across PubMed/MEDLINE, Scopus, Web of Science, and Google Scholar utilizing various combinations of terms related to parasitology, *in silico* techniques, computational genomics, immunoinformatics, reverse vaccinology, molecular docking, molecular dynamics, ADMET prediction, artificial intelligence, machine learning, systems biology, and host–parasite interactions. The search was limited to articles published in English that focused on parasite biology, target discovery, vaccine development, drug discovery, structural biology, and computational modeling.

The reference lists of the selected articles and pertinent reviews were examined for additional resources specific to the domain. Studies were included if they discussed computational methods applied to parasitic organisms or diseases; papers that concentrated exclusively on non-computational experimental methods were not considered. Given that this review is narrative in nature, the search strategy emphasized thematic breadth and coverage rather than adhering to a strictly systematic selection process.

## 3 Computational genomics and gene prediction

The cornerstone of contemporary *in silico* parasitology lies in the availability of complete or nearly complete parasite genome sequences. Sequencing has provided a strong molecular framework and generated genomic and transcriptomic datasets analyzed mainly with bioinformatics tools (15). In parasitic nematodes and protozoa, next-generation sequencing, together with assembly, annotation, and differential expression pipelines, has revealed genes linked to host invasion, immune evasion, and drug resistance. However, the AT-rich and highly repetitive structure of many parasite genomes creates major challenges for precise gene identification and functional annotation, making advanced computational tools essential (15, 16).

High-throughput sequencing has broadened access to genomics and improved understanding of parasitic diseases (17). Key parasite genomes, including *Plasmodium falciparum*, *Trypanosoma brucei, Leishmania major, Schistosoma mansoni,* and *Toxoplasma gondii*, have provided a strong framework for systematic bioinformatic analysis (18–21). Genome sequencing also helps construct phylogenetic trees and clarify evolutionary relationships among parasites and related species (22).

### 3.1 Gene prediction and functional annotation

Gene prediction is the process of identifying protein-coding regions and gene structures within raw genomic sequences. Ab initio tools such as AUGUSTUS, GeneScan, GLIMMER, and GeneMark are specifically designed for high AT-content and non-canonical splice sites. These tools utilize Hidden Markov Models (HMMs) to distinguish between coding and non-coding sequences by analyzing codon usage bias, splice site signals, and the lengths of open reading frames (ORFs). The performance of these tools is significantly enhanced when they are integrated with RNA-seq data, protein homology, and domain-based signals (23, 24). Nevertheless, these tools are heavily reliant on training data derived from model organisms and frequently encounter difficulties with parasite-specific characteristics, including atypical gene structures, short ORFs, and overlapping or alternatively spliced transcripts. This often results in the generation of fragmented or incomplete gene models, particularly in AT-rich and structurally divergent genomes.

Functional annotation assigns biological significance to predicted gene products through sequence homology searches utilizing BLAST against databases such as NCBI, UniProt, and TrEMBL. Numerous parasite genes—designated as ‘hypothetical proteins’ or ‘proteins of unknown function’—do not have identifiable homologs in non-parasitic organisms, underscoring the distinct evolutionary adaptations of these pathogens. Instruments like InterProScan forecast protein domains, motifs, and subcellular localization, providing functional insights into parasite-specific proteins that frequently act as promising, non-redundant drug targets (13, 25). Mistakes at this fundamental level can lead to errors in pathway reconstructions, essentiality predictions, and comparative analyses (26, 27).

The primary challenges associated with gene prediction involve fragmented draft assemblies that are marked by gaps and repetitive sequences, which result in misassembled alleles and incomplete gene models (12, 28). Additionally, the presence of non-canonical genes, overlapping transcripts, and intricate regulatory regions that are either inadequately represented or misannotated distorts comparative genomics and the reconstruction of metabolic networks (16, 29, 30). Furthermore, stage-specific expression profiles, trans-splicing, and polycistronic transcription are phenomena that conventional tools fail to adequately capture (24). Lastly, lineage-specific genes that exhibit limited homology to reference databases lead to a systematic underestimation of gene content (31).

Additionally, repetitive elements, multigene families, and antigenic variation systems further complicate prediction algorithms, leading to duplicated or collapsed gene models, while the majority of pipelines encounter difficulties in identifying small genes, non-coding RNAs, and regulatory elements influenced by epigenetic and chromatin factors (31). The integration of RNA-seq, proteomics, and cDNA sequencing enhances the reliability of annotations (32, 33), and functional validation through CRISPR-based editing substantiates that predicted ORFs impact parasite fitness (34, 35).

In summary, gene-prediction pipelines are essential for the large-scale annotation of parasite genomes, but they should be regarded as frameworks for generating hypotheses rather than as conclusive gene catalogs. The accurate interpretation of genomes still relies on integrative strategies that combine computational predictions with transcriptomic, proteomic, and functional experimentation to guarantee biological reliability and relevance in the discovery of drug and vaccine targets.

### 3.2 Comparative genomics and metabolic reconstruction

Comparative genomics is a robust *in silico* method that compares parasite genomes, and parasite genomes with those of non-parasitic organisms, to identify conserved and unique genetic components (15). It can identify genes essential for parasite survival across species, making them promising targets for broad-spectrum drug development. It also reveals genes gained or lost during parasitism, providing insight into host adaptation and pathogenesis (13).

**Figure 1:**
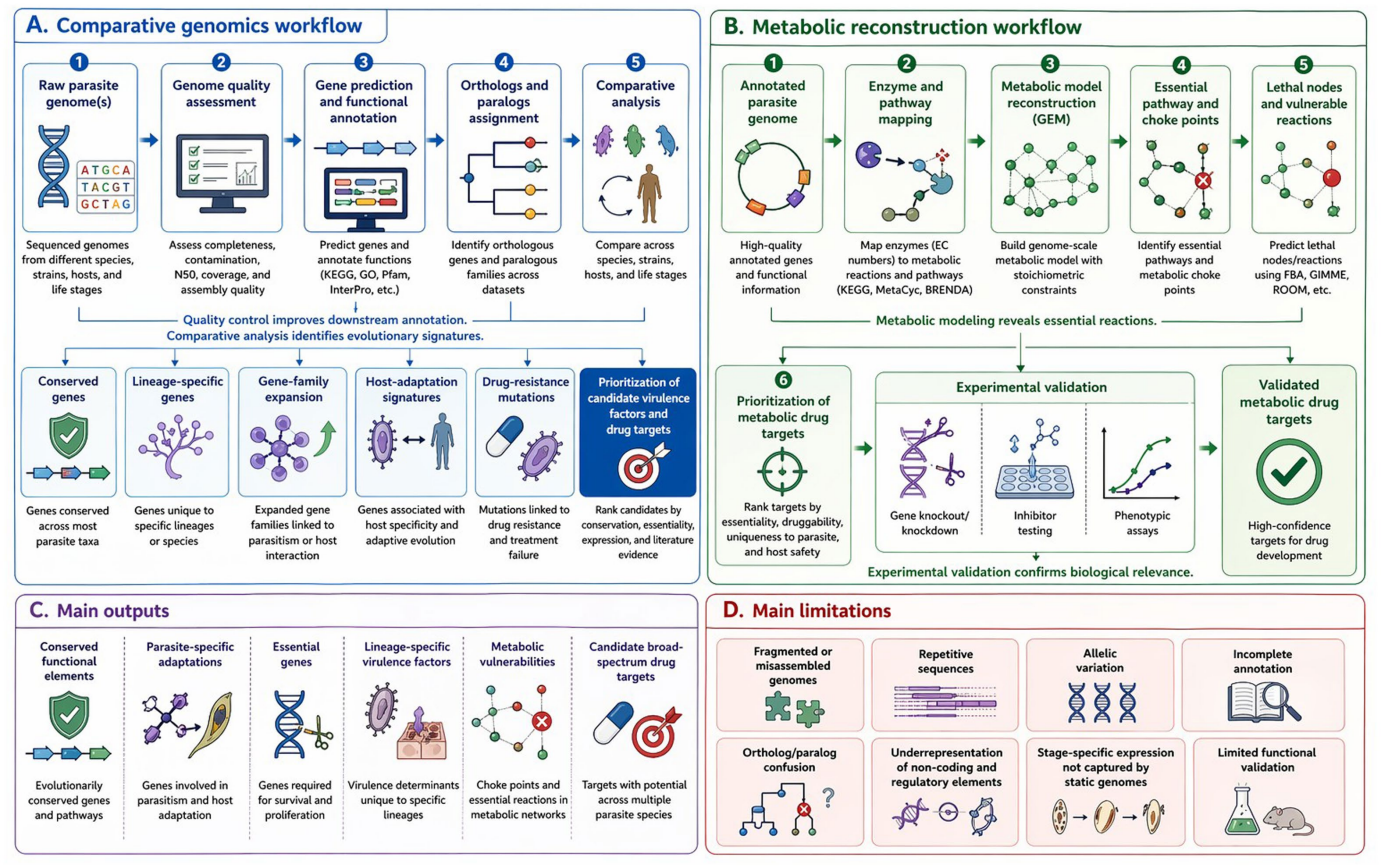
Comparative genomics and metabolic reconstruction. **(A)** Comparative genomics workflow; **(B)** Metabolic reconstruction workflow; **(C)** Main outputs; **(D)** Main limitations.

A valuable application of comparative genomics is the reconstruction of genome-scale metabolic models (GEMs), which use genomic data to predict the full set of biochemical reactions an organism can perform (13). This helps identify critical metabolic pathways and choke points, where inhibition would be lethal to the parasite, making them strong drug targets (13), Figure 1. Comparative genomics enables systematic comparison of parasite genomes across species, strains, hosts, and life stages. It reveals evolutionary patterns, conserved functional elements, and lineage-specific adaptations. It has been widely used in *Plasmodium*, *Leishmania*, *Trypanosoma*, *Giardia*, and helminths, helping identify gene-family expansions, host-adaptation signatures, drug-resistance mutations, and distinctive metabolic traits (36–38).

However, the method has important limitations. Its accuracy depends on the completeness and quality of genome assemblies, which are often fragmented, misassembled, or poorly annotated in non-model and neglected parasites. Repetitive sequences, allelic variation, and complex gene structures can distort orthology assignments and selection signals, leading to false positives or missed parasite-specific loci (39–41). High genetic diversity and rapid evolution also make it difficult to distinguish orthologs from paralogs, especially in expanded gene families linked to antigenic variation or host interaction. This increases the risk of missing lineage-specific virulence factors or novel genes without recognizable homologs (42).

Functional interpretation is another limitation. Comparative genomics often depends on sequence similarity, but conserved sequence does not always mean conserved function, especially in parasites with strong regulatory, metabolic, and environmental adaptation (43, 44). Many studies focus on coding regions and underrepresent non-coding RNAs, regulatory sequences, and epigenetic modifications that strongly influence parasite biology (45, 46). Static genomic data also fails to capture stage-specific gene expression, transcriptional regulation, and host-dependent phenotypes (47, 48). Horizontal gene transfer, hybridization, and genome plasticity, common in some protozoa and helminths, can further obscure evolutionary relationships and complicate phylogenetic inference (49, 50).

Integrating comparative genomics with transcriptomic, proteomic, and metabolomic data, along with targeted gene knockout or knockdown studies, helps test whether candidate genes and pathways have true phenotypic relevance, such as effects on growth, infectivity, or drug sensitivity. These studies show that comparative genomics narrows the search space for functional analysis, but many computationally identified candidates do not prove biologically significant under physiological conditions (51).

## 4 Immunoinformatics and vaccine design

**Figure 2:**
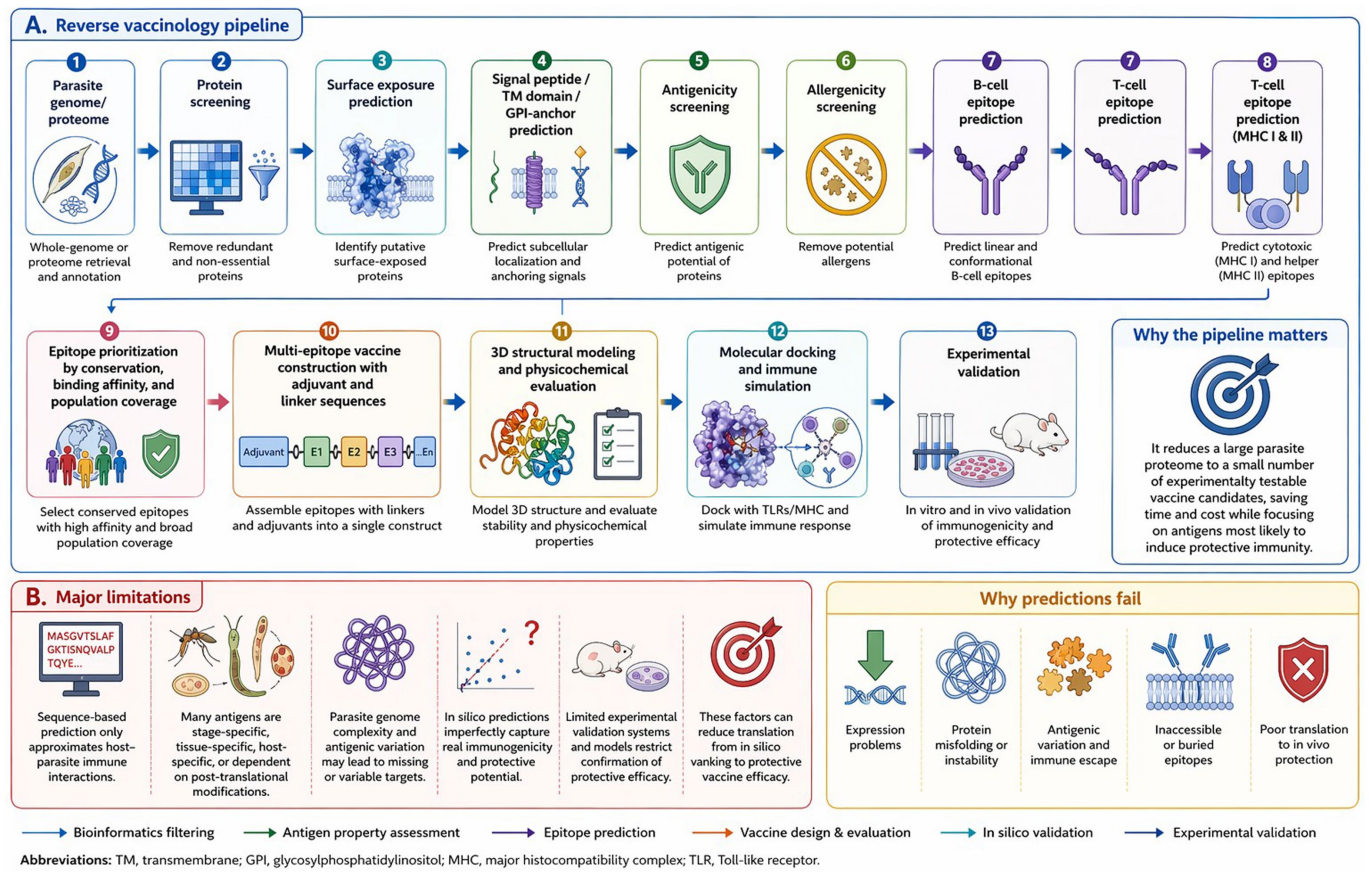
Immunoinformatics and reverse vaccinology for parasitic vaccine design. **(A)** Reverse vaccinology pipeline; **(B)** Major limitations. TM, transmembrane; GPI, glycosylphosphatidylinositol; MHC, major histocompatibility complex; TLR, Toll-like receptor.

Developing vaccines against parasitic diseases is difficult because parasites have complex life cycles, strong antigenic variability, and effective immune evasion mechanisms. Immunoinformatics provides a systematic, high-throughput framework for vaccine development through reverse vaccinology, which starts from the parasite genome or proteome and computationally ranks the most promising vaccine candidates before experimental testing (52, 53), Figure 2.

### 4.1 Reverse vaccinology pipeline

A standard *in silico* reverse vaccinology pipeline involves several steps. Genome-wide screening identifies proteins likely to be surface-exposed and accessible to host immune effectors by predicting signal peptides, transmembrane domains, or GPI anchors (54). Antigenicity and allergenicity tools such as VaxiJen and AlgPred evaluate whether candidates can induce immune responses without adverse reactions (55). Epitope prediction is the central step: B-cell epitopes are identified using ABCpred and ElliPro, while T-cell epitopes are predicted using NetMHCpan and the Immune Epitope Database (IEDB), which assess peptide–MHC binding affinity and help predict CD4 + and CD8 + T-cell activation important for defense against intracellular parasites (1, 53). Specialized resources such as MHC-Ld-DB and LDEP-DB support leishmaniasis vaccine studies by providing curated *Leishmania* peptides across host MHC alleles, which is important given population-level immune diversity. Promising epitopes are then combined into multi-epitope subunit vaccine constructs, linked with suitable adjuvants and linkers, and modeled in three dimensions with physicochemical properties assessed using tools such as ExPASy ProtParam (56).

These computational filters, including subcellular localization, signal peptides, epitope density, antigenicity, allergenicity, conservation, and MHC binding potential, help identify promising antigens from large datasets. However, the pipeline has important limitations because of both computational simplification and parasite biology (57).

A key limitation is that Reverse vaccinology depends heavily on sequence-based prediction, which only approximates the complexity of host–parasite immune interactions. Many parasite antigens are stage-specific, host-specific, tissue-specific, or dependent on post-translational modifications such as glycosylation or phosphorylation, and these features are poorly represented in standard *in silico* filters. As a result, transient, conformation-dependent, or condition-specific proteins may be missed entirely (58). This problem is even greater in parasites with complex life cycles, such as *Plasmodium*, *Schistosoma*, *Leishmania*, and *Ascaris*, where protective immunity depends on life stage, adjuvant formulation, and host context rather than sequence alone (55, 59).

Another major limitation is genome complexity. The large, repetitive, and genetically diverse genomes of many eukaryotic parasites make accurate annotation difficult and increase the chance of missing or mispredicting vaccine targets (60, 61). Antigenic variation, in which parasites change their surface proteins over time, also reduces the durability of immune protection against single antigens (62).

In addition, predictions of antigenicity or MHC binding do not always match real immunogenicity or protective efficacy. Even conserved and apparently immunogenic antigens may fail experimentally because of poor expression, incorrect folding, or limited access to immune effectors (63). Validation is further constrained by the lack of robust experimental systems for many parasites, especially when culture systems or accurate infection models are difficult to establish (64, 65).

## 5 *In silico* drug discovery and development

**Figure 3:**
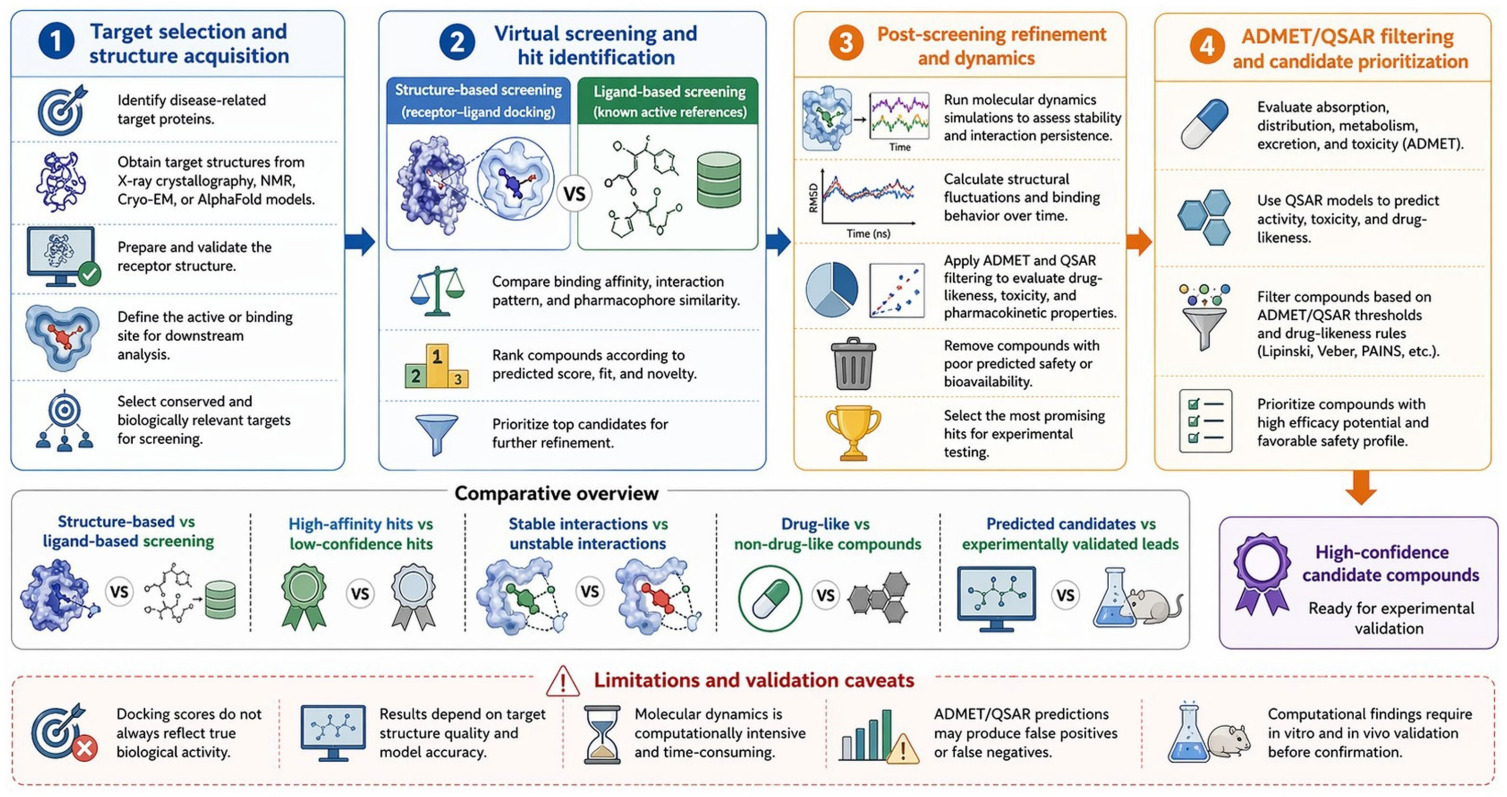
Integrated structure- and ligand-based drug discovery workflow for candidate prioritization.

The search for new antiparasitic drugs remains difficult because parasites adapt rapidly and drug resistance continues to emerge. *In silico* drug discovery offers rational, high-throughput alternatives to traditional screening and includes two main strategies: structure-based drug design (SBDD), which uses three-dimensional target protein structures, and ligand-based drug design (LBDD), which uses known activity profiles of existing compounds (4), Figure 3.

### 5.1 Virtual screening and molecular docking

Virtual screening examines large chemical libraries to identify compounds most likely to bind a target. Molecular docking is the main method and predicts the best binding orientation and affinity of a ligand in the target site. Common tools such as AutoDock Vina, Glide, and GOLD simulate drug-target interactions in four steps: obtaining the target structure experimentally or by prediction methods such as AlphaFold, I-TASSER, or SWISS-MODEL; preparing the chemical library; running docking simulations; and prioritizing hits by predicted binding affinity (4).

Parasite-focused inhibitor databases are central to these workflows. LeishInDB contains more than 7,000 *Leishmania* inhibitors, TrypInDB contains more than 14,000 *Trypanosoma* inhibitors, and Malariamdb ChemDB centralize compound libraries, target annotations, and chemical property data to support hit identification and lead optimization (66, 67). A notable example is the discovery of novel inhibitors of *Leishmania donovani* trypanothione reductase (LdTR) through FDA-approved drug repurposing screens, which can reduce both development time and cost (4, 68). Limitations include the lack of high-resolution parasite target structures, variation in scoring functions across platforms, and the fact that docking often evaluates interactions under static and idealized conditions that do not reflect the dynamic parasite environment (66).

### 5.2 Molecular dynamics and ADMET prediction

While molecular docking offers a static representation of the drug-target complex, Molecular Dynamics (MD) simulations provide a dynamic perspective (4). MD simulation is a computational technique that models the time-dependent physical movements of atoms and molecules by solving Newton’s equations of motion. It simulates how proteins, ligands, water, ions, lipids, and membranes behave over timescales from picoseconds to microseconds, providing a dynamic, time-resolved view of molecular systems that goes beyond static structures from X-ray crystallography, NMR, or docking. This information is vital for validating the outcomes of molecular docking and enhancing lead compounds.

In parasitology, MD is routinely applied to study parasite proteins such as *P. falciparum aquaporin (PfAQP),* plasmopsins aspartic proteases*, L. donovani* inositol phosphorylceramide synthase (IPCS), trypanosomes glycosomal enzymes, and helminth tubulin (69, 70). Software like GROMACS, AMBER, NAMD, Desmond, and OpenMM simulate protein-ligand stability, channel gating, resistance mutations, binding pocket flexibility, and host–parasite interactions after docking. These studies support drug discovery workflows for malaria, leishmaniasis, trypanosomiasis, and helminths by validating hits and refining leads (69, 71–73).

MD captures protein flexibility, induced-fit effects, water-mediated hydrogen bonds, hydrophobic contacts, salt bridges, and lipid interactions that rigid docking cannot model, making it ideal for dynamic parasite membrane proteins, stage-specific targets, and allosteric sites. It quantifies binding stability through root-mean-square deviation (RMSD), root-mean-square fluctuation (RMSF), radius of gyration, and free energy calculations like MM/GBSA or MM/PBSA, improving lead prioritization and reducing false positives (71). MD revealed asynchronous water/glycerol transport in PfAQP tetramer channels during malaria studies, confirmed IPCS inhibitor stability in *Leishmania*, explained glycosomal glycolysis chokepoints in trypanosomes, and showed how resistance SNPs alter pocket geometry in helminth tubulin. MD also supports resistance prediction by comparing wild-type vs. mutant trajectories and enables binding free energy profiling across compound series (74).

Extensive MD simulations on *Leishmania* spp. have focused on trypanothione reductase (LdTR), sterol C-24 methyltransferase (LdSCMT), and pteridine reductase 1 (PTR1), all of which are drug targets not found in humans. A microsecond-scale simulation of the LdTR–nitazoxanide complex showed stable NADPH-binding site engagement and revealed a novel allosteric inhibition site near the catalytic dyad. Additionally, MD trajectories highlighted that LdSCMT has a substrate-binding channel about 3 Å narrower than its human counterpart, explaining the efficacy of azole-class compounds against *Leishmania* while protecting human cholesterol biosynthesis (70, 75, 76). MD simulations have been pivotal in *P. falciparum* research, aiding in the characterization of drug-resistance mechanisms at an atomic level. Simulations of mutant Kelch13 propeller domains with artemisinin-resistance mutations (C580Y, R539T, M476I) revealed reduced flexibility of the *β*-propeller scaffold and altered electrostatic surfaces, providing a basis for reduced drug activation. Additionally, MD-based binding free-energy calculations using MM/PBSA and MM/GBSA methods have effectively ranked inhibitors of PfDHFR and PfDHPS, accurately predicting the selectivity for pyrimethamine. Furthermore, MD studies have facilitated the optimization of spiroindolone PfATP4 inhibitors, including KAE609, by demonstrating induced-fit changes in the ion-channel cavity that are not evident in crystal structures (77–79). Simulations of *T. brucei* phosphoglycerate kinase (TbPGK)—compartmentalized inside glycosomes, which have no mammalian equivalent—demonstrated that substrate binding induces a 12° hinge-bending domain closure, and that bisubstrate analogs locking the enzyme in the open conformation inhibit the parasite glycolytic flux at nanomolar concentrations without affecting cytosolic human PGK. For *T. cruzi*, as described in Section 10.3, microsecond MD confirmed the stable accommodation of nilotinib in the DHFR-TS folate-binding pocket, providing the computational evidence needed to advance this BCR-ABL repurposed compound into *in vitro* validation (80, 81). Helminth research has also employed coarse-grained MD—using MARTINI force-field representations that reduce computational expense by grouping atoms into beads—to simulate the membrane dynamics of excretory-secretory proteins anchored to larval tegument surfaces, enabling prediction of antibody-accessible epitope exposure across helminth life stages (82, 83).

Despite their power, MD simulations face several significant limitations in the parasitological context. First, simulation timescales remain far shorter than biologically relevant processes: most antiparasitic targets undergo slow conformational rearrangements, allosteric communication events, or drug-induced protein unfolding over milliseconds to seconds—timescales that are computationally inaccessible even with GPU acceleration and enhanced sampling methods (82). This limitation is especially acute for multi-domain parasite proteins such as the *Plasmodium* Hsp90 chaperone and the *Trypanosoma* editosomes, where interdomain communication governs drug sensitivity but is invisible within sub-microsecond simulation windows (84).

MD accuracy depends heavily on starting structure quality, but parasite proteins often require homology models or AlphaFold predictions with errors that propagate through simulations, especially for divergent sequences lacking close templates. Computational demands are extreme—100-500 ns production runs for membrane-embedded targets require weeks on GPU clusters—and accessible timescales miss slow conformational changes, life-cycle transitions, or rare binding/unbinding events critical to parasite biology. Force fields (CHARMM36, AMBER ff14SB, OPLS-AA) poorly parameterize parasite-specific glycosylation, lipidation, heme groups, and membrane compositions, leading to solvation artifacts and inaccurate dynamics. Simulations oversimplify *in vivo* conditions by excluding host immune factors, compartmentalization, pH gradients (e.g., *Leishmania* phagolysosomes), and adaptive metabolic responses. Results vary across software and force fields, requiring careful benchmarking, and all predictions demand experimental validation through enzyme assays, parasite growth inhibition, and animal models (85).

### 5.3 Natural products and AI-driven discovery

The conventional application of natural products for the treatment of parasitic diseases is undergoing modernization through *in silico* methods. Computational techniques are now employed to examine extensive databases of natural compounds for their anti-parasitic properties (86). Artificial intelligence and machine learning models are developed using existing drug-parasite interaction data to forecast new active compounds, frequently uncovering novel scaffolds and mechanisms of action that traditional screening methods might overlook (86). This collaboration between natural product chemistry and computational biology presents a promising pathway for revitalizing the anti-parasitic drug pipeline.

A primary limitation is the reliance on precise and comprehensive structural data. High-resolution experimental structures are frequently not accessible for parasite targets, necessitating the use of homology models or predicted structures. Any inaccuracies or uncertainties in these templates can propagate through processes such as docking, pharmacophore mapping, molecular dynamics simulations, and binding-energy calculations, thereby diminishing confidence in the predicted interactions. Furthermore, insufficient conformational sampling and an oversimplified depiction of protein flexibility often result in the omission of induced-fit effects, transient conformations, and long-timescale dynamics. These challenges are especially evident in parasites, where proteins may exhibit unique folds, species-specific active sites, and stage-dependent expression—elements that significantly affect binding yet are challenging to model computationally (87, 88).

Another limitation is found in the oversimplified approach to biological complexity. Docking and scoring workflows generally assess drug–target interactions under idealized circumstances, neglecting factors such as compartmentalization, host–parasite interactions, and cofactor requirements that influence drug efficacy in vivo. Consequently, compounds that are predicted to bind strongly *in silico* may turn out to be inactive in parasite cells or animal models. Even compounds with outstanding binding scores may not succeed due to poor solubility, low uptake, rapid metabolic clearance, or toxicity to host cells. While *in silico* ADMET tools can identify problematic molecules at an early stage, they are still inadequate in capturing the complete complexity of host and parasite pharmacology. Furthermore, most computational models do not fully account for parasite-specific characteristics such as stage variation, adaptive metabolism, or the emergence of drug resistance. Since resistance often develops through genetic mutations or pathway rewiring, static models that lack evolutionary dynamics may overestimate the long-term efficacy of compounds (87).

Validation continues to be a significant obstacle. Predicted hits require confirmation through enzyme-inhibition assays, parasite-growth experiments, cytotoxicity testing in host cells, and, ideally, infection models that assess efficacy under physiological conditions. A compound may inhibit a purified enzyme yet fail in the intact parasite due to inadequate uptake, alternative pathways, or unfavorable bioavailability. Consequently, the most effective discovery pipelines are hybrid frameworks: virtual screening and docking prioritize candidates; molecular dynamics and ADMET refine the shortlist; and experimental assays ascertain genuine antiparasitic potential.

Ultimately, *in silico* drug discovery necessitates considerable computational infrastructure, standardized datasets, and interdisciplinary expertise—resources that are frequently scarce in regions most impacted by parasitic diseases. Coupled with data-quality challenges, the absence of experimental reference systems, and the complexities of modeling parasite–host biology, these elements hinder translational success. In summary, *in silico* methods offer robust hypothesis-generating and prioritization tools but must be supplemented by thorough experimental validation and biological integration to guarantee true therapeutic relevance in parasitology.

## 6 Modeling host–parasite interactions

**Figure 4:**
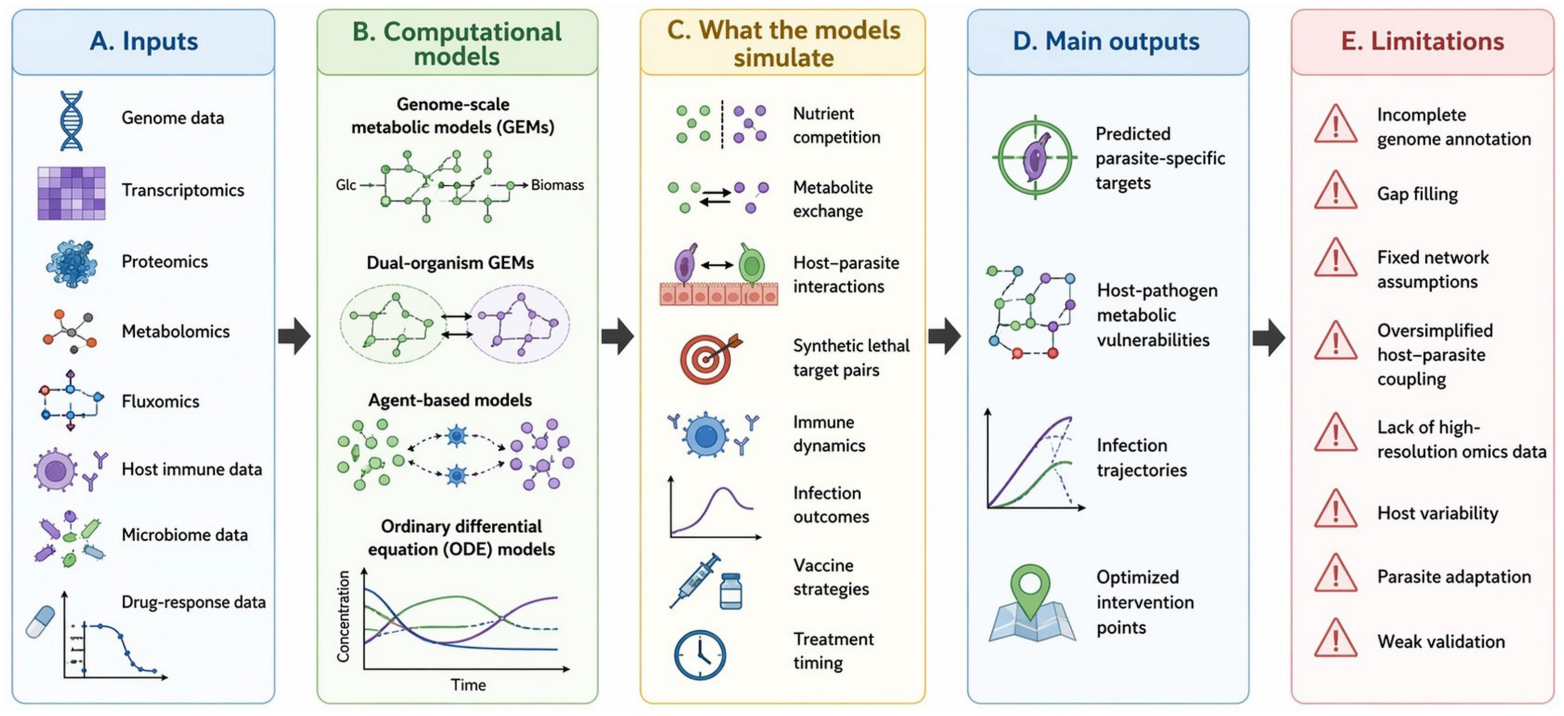
Systems biology modeling of host–parasite interactions. Computational models integrate multi-omics and host immune data to simulate parasite metabolism, nutrient exchange, immune dynamics, and drug response. GEMs and dual-organism GEMs identify metabolic vulnerabilities and synthetic lethal targets, while agent-based and ODE models explore infection outcomes and treatment strategies. However, model accuracy is limited by incomplete data, simplified assumptions, and insufficient experimental validation. **(A)** Inputs; **(B)** Computational models; **(C)** What the models simulate; **(D)** Main outputs; **(E)** Limitations.

Parasitism represents a fundamental interaction between two separate biological entities: the host and the parasite, each possessing its own intricate molecular machinery. Grasping this complex dialogue is essential for creating interventions that can either interrupt the life cycle of the parasite or enhance the immune response of the host. *In silico* techniques, especially those based in systems biology, are particularly well equipped to model this complexity (89), Figure 4.

### 6.1 Protein–protein interaction (PPI) networks

Parasite survival frequently depends on hijacking host cellular pathways through specific protein–protein interactions (PPIs). Computational PPI prediction employs sequence-based methods (domain–domain interactions and co-evolutionary signals), structure-based approaches (docking host and parasite protein structures to predict interaction interfaces), and integrative strategies combining genomic, transcriptomic, and proteomic data—including dual RNA-seq experiments that simultaneously profile host and parasite transcriptomes during active infection. Analysis of predicted PPI networks identifies hub proteins—parasite proteins engaging multiple host partners—that are essential for pathogenesis and represent high-priority drug targets. In filarial diseases, for example, *in silico* PPI predictions have elucidated how infective larvae modulates host immunosuppressive signaling to establish chronic, immunotolerant infection (89, 90).

### 6.2 Systems biology and metabolic modeling

Systems biology employs computational models to examine emergent behavior of the entire host–parasite system. GEMs expanded to represent host–parasite metabolic interactions can simulate competition for nutrients, metabolite exchange, and the cascading effects of inhibiting parasite-specific metabolic steps on host physiology. Dual-organism GEMs facilitate identification of synthetic lethal target pairs—gene combinations whose simultaneous inhibition is selectively lethal to the parasite—enabling more precise drug development. Agent-based and ordinary differential equation (ODE) models of host immune dynamics enable simulation of infection outcomes, comparative vaccine strategies, and optimal treatment timing (13, 89).

First, many models rely on incomplete or biased datasets, especially for parasites with poorly annotated genomes or missing biochemical pathways. This often necessitates “gap filling,” which can lead to networks that do not accurately reflect true cellular or physiological processes (91). Additionally, these models typically assume a steady-state metabolism or a fixed network structure, ignoring the dynamic nature of biological systems, such as temporal changes, regulatory responses, and different stages of the parasite’s life cycle. This simplification makes it difficult to capture stage-specific phenomena like antigenic switching, immune evasion, or developmental differentiation (92).

Second, the metabolic coupling between the host and parasite is often oversimplified. Most models treat them as loosely connected systems, which neglects the complexities of metabolite exchange, transport kinetics, and spatial compartmentalization across tissues and organelles. This is especially problematic in intracellular infections, where membrane transport processes and spatial limitations significantly influence metabolic fluxes. The lack of high-resolution experimental data—such as metabolomics, fluxomics, transcriptomics, and proteomics—further hampers the calibration and validation of these models, leading to reliance on inferred or estimated parameters that reduce confidence in their predictions (93, 94).

Third, host variability and adaptive parasite responses are rarely incorporated into current models. Variations in immune responses, genetic backgrounds, microbiome composition, diet, and physiological conditions can greatly influence the metabolic and immune landscape, but most models assume an “average” host. Moreover, regulatory mechanisms like transcriptional control, enzyme kinetics, signaling pathways, and post-translational modifications are generally excluded, limiting the models’ ability to simulate genuine adaptive behaviors. Parasites can alter their metabolic networks in response to host immunity or drug exposure, activating alternative pathways or entering dormant stages—these dynamic adaptations are difficult to represent in static models. The complexity of drug resistance mechanisms and the challenges of integrating multi-omics and multi-scale data further restrict the scalability and predictive power of these models. Overall, while systems biology modeling provides valuable frameworks, its results should be viewed as approximations requiring extensive experimental validation (95–97).

## 7 AI and machine learning in parasitology

**Figure 5:**
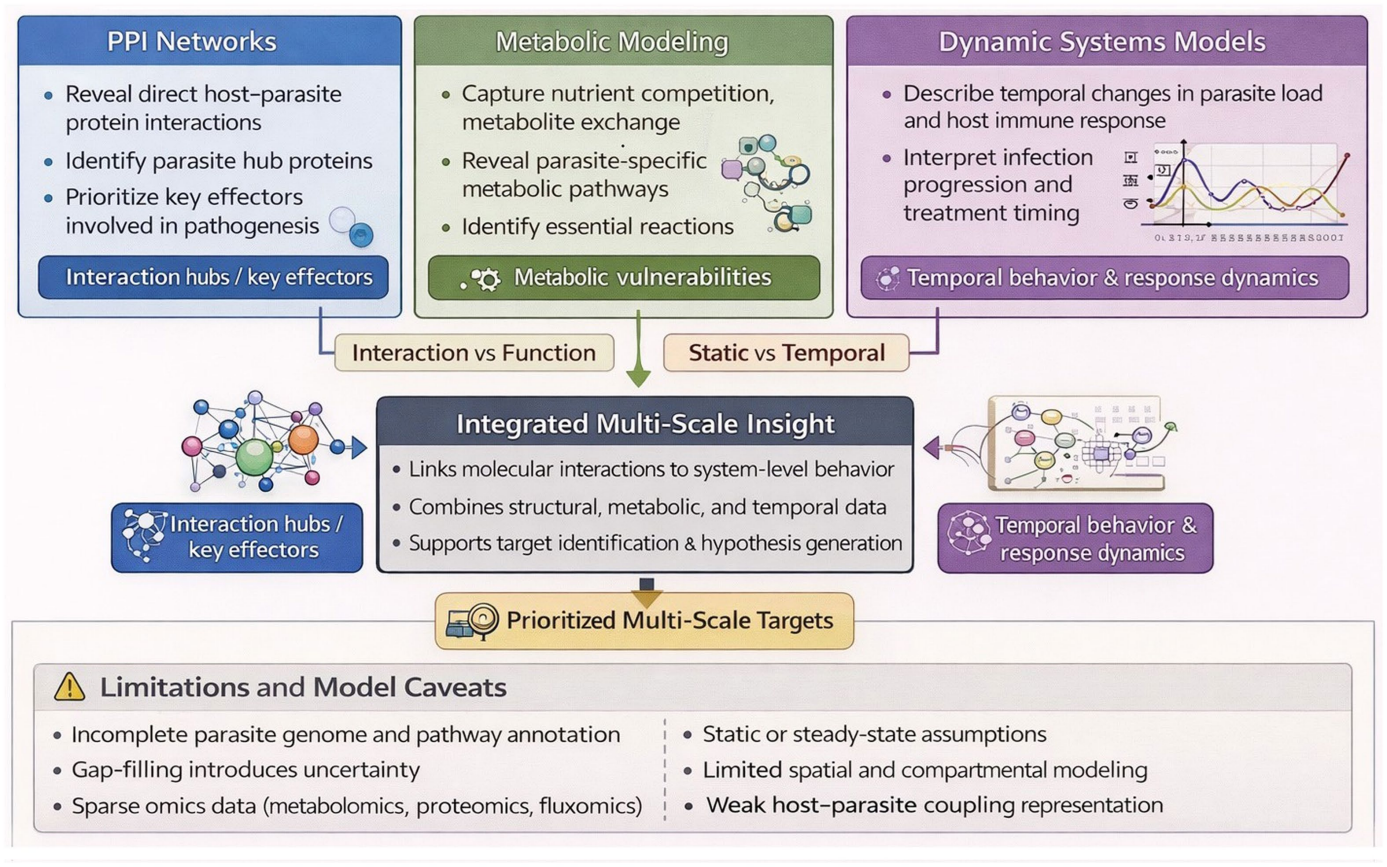
Comparative multi-scale modeling of host–parasite interactions. PPI networks, metabolic modeling, and dynamic systems approaches provide complementary insights into molecular interactions, metabolic dependencies, and infection dynamics. Their integration enables identification or key vulnerabiilities.

The recent increase in computational capabilities and the advancement of complex algorithms have established Artificial Intelligence (AI) and Machine Learning (ML) as revolutionary elements in the field of parasitology. These technologies are particularly adept at recognizing patterns and making predictions within vast and intricate datasets, providing solutions in various domains, including diagnostics and the prediction of drug resistance, Figure 5.

### 7.1 Automated diagnostics and image analysis

One of the most immediate uses of artificial intelligence in the field of parasitology is the implementation of automated microscopy-based diagnostics—especially for malaria, where the precise identification of infected erythrocytes in blood smears is crucial for saving lives, yet traditionally necessitates the skills of an expert microscopist. Deep learning models—particularly Convolutional Neural Networks (CNNs)—that are trained on extensive annotated image datasets can automatically detect and segment red blood cells, recognize infected cells, classify parasite species and their intraerythrocytic developmental stages (such as ring, trophozoite, and schizont), and compute parasitemia levels. Research has shown that AI-driven systems can achieve diagnostic accuracy that is comparable to or even surpasses that of expert microscopists, thus providing scalable solutions for settings with limited resources. Similar methodologies are being applied to other parasitic organisms in the realms of microscopy, histopathology, and whole-slide imaging.

AI-based diagnostics nonetheless face important unresolved challenges. Training dataset scarcity and annotation quality are primary bottlenecks: labeling parasitic forms requires specialist expertise, making large-scale, geographically diverse dataset construction costly and time-intensive. Data heterogeneity—differences in microscope type, staining protocols, magnification, illumination, and sample preparation—degrades model robustness across sites. Many AI systems demonstrate high internal accuracy but underperform substantially on independent external datasets due to overfitting and inadequate external validation due to variations in imaging conditions, biological diversity, or geographic strains. Furthermore, models are particularly sensitive to noisy or degraded images that contain debris, overlapping structures, or background artifacts that are prevalent in authentic diagnostic samples. Deep learning models frequently function as ‘black boxes,’ generating predictions without transparent mechanistic reasoning and undermining clinician trust.

The majority of models do not account for pertinent clinical or ecological metadata—such as patient history, the life-cycle stage of parasites, co-infections, or environmental influences—that are vital for accurate diagnostic interpretation. The ongoing evolution of parasites, their phenotypic plasticity, and the emergence of new strains can also impair performance if models are not regularly retrained with updated datasets. While integrating multi-omics, clinical, and epidemiological data could enhance contextual understanding, it remains a technically complex and computationally intensive task. These limitations highlight the necessity for standardized data acquisition processes, transparent AI frameworks, and stringent clinical validation protocols. Accordingly, AI is currently most appropriately deployed as a decision-support tool augmenting expert human diagnostics, rather than as an autonomous diagnostic system (98, 99).

### 7.2 Predictive modeling for drug resistance and epidemiology

Machine learning models that are trained on genomic data derived from phenotypically characterized drug-sensitive and drug-resistant strains of parasites can detect genetic markers—such as SNPs, copy number variations, and gene amplifications—that are linked to resistance. This capability facilitates swift *in silico* susceptibility profiling of newly sequenced clinical isolates, thereby aiding treatment decisions and enhancing public health surveillance (100). Artificial Intelligence is increasingly being incorporated into mathematical epidemiological models to enhance the prediction of disease outbreak dynamics and transmission patterns. By integrating satellite-derived climate and land-use information, vector distribution maps, human mobility trends, and indicators of health system capacity, machine learning models are capable of producing more precise, spatially detailed forecasts of disease transmission risk (100).

Predictive models are constrained, however, by data scarcity, inconsistency, and geographic bias; surveillance information from endemic regions is frequently incomplete or unevenly distributed, characterized by temporal gaps and underreporting of resistant strains. Consequently, parameter estimates—such as transmission rates, mutation frequencies, and treatment compliance—can be uncertain, leading to broad confidence intervals and unstable predictions. Model outputs should be communicated with transparent uncertainty quantification to support—rather than supplant—expert epidemiological judgment.

The intricacy and diversity of parasite systems pose additional challenges to the generalizability of models. The mechanisms underlying resistance evolution and disease transmission differ significantly across various regions, host species, and environmental contexts, indicating that a model designed for one situation may not yield effective results in another. The swift genetic adaptation of parasites, along with the emergence of new resistance mechanisms, can rapidly make models outdated unless they are regularly updated with the latest genomic and epidemiological information. Validation is crucial but often presents challenges. However, logistical, ethical, and temporal limitations frequently hinder systematic testing, especially for long-term predictions.

In conclusion, the primary benefit of predictive modeling is directing surveillance priorities, investigating feasible scenarios, and formulating hypotheses for empirical fieldwork and laboratory research to test.

### 7.3 AlphaFold and structural biology

AlphaFold, developed by DeepMind, represents a groundbreaking advancement in structural biology, particularly impacting the field of parasitology. This technology, along with similar innovations, is capable of predicting three-dimensional protein structures with a precision that closely resembles experimental results, based solely on amino acid sequences. This capability eliminates a significant barrier in structure-based drug design (SBDD) and the development of vaccines. Consequently, researchers now have access to high-quality structures for thousands of parasite proteins, including those that are difficult to crystallize, thereby significantly accelerating target validation and virtual screening (13).

The advent of AlphaFold and similar structural biology algorithms has transformed the field of parasitology by facilitating the creation of high-quality three-dimensional models for thousands of parasite proteins that are challenging or impossible to crystallize. These predictions have expedited functional annotation, structure-based drug discovery, and antigen identification. Nevertheless, despite their advantages, AlphaFold models are subject to significant limitations that must be carefully evaluated within parasitic systems.

A key limitation is that AlphaFold produces a single, static conformation based exclusively on sequence data and does not take into account cofactors, ligands, metal ions, prosthetic groups, or post-translational modifications. Numerous parasite enzymes and structural proteins depend on heme groups, metal centers, or host-derived cofactors and the lack of these components can alter the electrostatics and geometry of active sites or interaction surfaces. As a result, docking studies that utilize AlphaFold outputs may not accurately reflect the biologically relevant ‘complexed’ or ‘drug-bound’ state. This limitation also applies to dynamic or stage-specific proteins whose conformations vary throughout the life cycle of the parasite.

Another limitation is that AlphaFold generally models isolated domains or monomeric units; however, numerous parasite proteins operate as multimers, membrane-embedded assemblies, or complexes that necessitate chaperones, lipid environments, or host-specific partners. These quaternary structures are frequently misrepresented or entirely absent in predictive models, which necessitates the use of complementary experimental techniques such as cryo-electron microscopy, X-ray crystallography, or co-purification from native parasites.

Furthermore, AlphaFold does not inherently predict protein function, catalytic activity, or ligand specificity, indicating that structural information must be combined with biochemical, functional, and omics data for accurate interpretation. Ultimately, connecting predicted structures with other biological layers is a complex and computationally intensive task. Merging structural predictions with genomics, metabolomics, and host–parasite interaction networks is crucial for contextualizing molecular mechanisms, yet it necessitates sophisticated data harmonization and cross-platform modeling. Collectively, these constraints highlight that AlphaFold and similar algorithms offer robust structural hypotheses rather than conclusive answers. Their predictions ought to be regarded as initial points—informing experimental design, mutagenesis, and inhibitor screening—but must undergo validation and refinement through experimental structural biology and functional studies in the field of parasitology.

### 7.4 Computational resources for *in silico* parasitology

The parasitology research ecosystem is supported by specialized databases, analytical platforms, and modeling environments, that enable integrated *in silico* investigation from genomics through epidemiology. Table 1 provides a consolidated reference of key tools, their primary applications, principal parasite groups covered, and salient limitations.

**Table 1:** Key computational resources for i*silico* parasitology research.

Category	Key tools/resources	Parasite group(s)	Main applications	Key limitations	Reference/URL	References
Genomic databases	EuPathDB, PlasmoDB, TriTrypDB, WormBase Parasite, GenBank, Ensembl Protists	All major parasites	Genome assembly, annotation, comparative genomics	Incomplete assemblies; annotation errors; rare parasite underrepresentation	https://veupathdb.org/veupathdb/app https://plasmodb.org/plasmo/app https://tritrypdb.org/tritrypdb/app https://veupathdb.org/ http://protists.ensembl.org/ https://parasite.wormbase.org/index.html https://www.ncbi.nlm.nih.gov/genbank/	Deroost et al. (101), Warrenfeltz et al. (102), Shanmugasundram et al. (103), Sternberg et al. (104)
Drug discovery databases	LeishInDB, TrypInDB, Malariamdb ChemDB, PubChem, ChEMBL	*Leishmania, Trypanosom, Plasmodium*	Inhibitor screening; lead optimization; drug repurposing	Limited coverage; inconsistent activity data; no *in vivo* validation	http://leishindb.biomedinformri.com/ https://trypindb.biomedinformri.com/ http://www.malarimdb.org/	Deroost et al. (101),Vijayakumar et al. (67, 105)
Immunoinformatics	MHC-Ld-DB, LDEP-DB, IEDB, NetMHCpan, VaxiJen	*Leishmania*	Epitope prediction; reverse vaccinology; vaccine design	Poor immune correlation; MHC gaps; ignores glycosylation	https://mhcfisld.biomedinformri.com/	Doytchinova and Flower (106), Ranjan et al. (107), Vijayakumar et al. (108), Vita et al. (109)
Transcriptomics	HISAT2, StringTie, DESeq2, Salmon	All parasites	Stage-specific expression; differential analysis	Limited life-cycle data; batch effects; normalization issues	https://github.com/DESeq2	Love et al. (110), Pertea et al. (111), Lim et al. (112)
Proteomics	UniProt, PRIDE, PeptideAtlas	All parasites	Protein ID; PTM mapping; functional annotation	Missing parasite proteins; poor validation	https://www.uniprot.org/	Apweiler et al. (113), Martens et al. (114), Deutsch (115)
Structural biology	AlphaFold3, SWISS-MODEL, PDB, ColabFold	All parasites	Structure prediction; docking preparation	Static models; poor dynamics; cofactor omission	https://alphafold.ebi.ac.uk/ https://swissmodel.expasy.org/ https://github.com/sokrypton/colabfold https://www.rcsb.org/ https://github.com/sokrypton/colabfold	Berman et al. (116), Schwede et al. (117), Mirdita et al. (118), Krokidis et al. (119)
Molecular docking	AutoDock Vina, Glide, GOLD, DOCK	All parasites	Virtual screening; hit identification	Scoring inaccuracies; ignores biological context	https://autodock.scripps.edu/ https://www.ccdc.cam.ac.uk/solutions/software/gold/	Trott and Olson (120), Reddy et al. (121)
Molecular dynamics	GROMACS, AMBER, NAMD, OpenMM	All parasites	Binding stability; conformational analysis	High computational cost; limited timescales	https://www.gromacs.org/ https://www.ks.uiuc.edu/Research/namd/ https://openmm.org/ https://www.amber.bio/	Phillips et al. (122), Pronk et al. (123), Eastman and Pande (124), Meyer et al. (125))
Metabolic modeling	COBRApy, KEGG, BioCyc, Recon 3D	*Plasmodium*, trypanosomatids	Flux analysis; pathway reconstruction	Incomplete pathways; steady-state assumptions	https://opencobra.github.io/cobrapy/ https://www.recon-3d.com/ https://biocyc.org/ https://www.genome.jp/kegg/	Kanehisa and Goto (126), (127); Ebrahim et al. Brunk et al. (128), Karp et al. (129)
Comparative genomics	OrthoMCL, OrthoFinder, BLAST+, MAFFT, IQ-TREE	All parasites	Orthology detection; evolutionary analysis	Divergent sequences; paralog confusion	https://orthomcl.org/orthomcl/app https://bio.tools/OrthoFinder https://mafft.cbrc.jp/ https://blast.ncbi.nlm.nih.gov/doc/blast-help/downloadblastdata.html http://iqtree.cibiv.univie.ac.at/	Li et al (130), Camacho et al. (131), Katoh and Standley (132), Emms and Kelly (133), Minh et al. (134)
Gene prediction	AUGUSTUS, GeneMark, GlimmerHMM	Non-model parasites	*De novo* annotation	Atypical gene structures; training bias	http://augustus.gobics.de/ https://exon.gatech.edu/ https://bio.tools/glimmer-hmm	Lukashin and Borodovsky (135), Stanke et al. (136), Allen et al. (137)
AI/ML platforms	TensorFlow, PyTorch, Scikit-learn, DeepChem	Diagnostic, resistance prediction	Image analysis; QSAR; diagnosis	Data scarcity; black-box nature; overfitting	https://www.tensorflow.org/ https://pytorch.org/ http://scikit-learn.org/ https://deepchem.io/	Rampasek and Goldenberg (138), Goscinski et al. (139), Liang et al. (140)
Epidemiological modeling	EpiModel (R), OpenMalaria, schemulator	Population-level	Transmission dynamics; intervention planning	Parameter uncertainty; oversimplified assumptions	https://bio.tools/epimodel https://github.com/SwissTPH/openmalaria https://validator.schema.org/	Yukich and Chitnis (141), enness et al. (142)
Network analysis	Cytoscape, STRING, OmicsNet, Gephi	Systems biology	PPI networks; multi-omics integration	Incomplete interactions; false positives	https://cytoscape.org/ https://string-db.org/ https://www.omicsnet.ca/ https://gephi.org/	Shannon et al. (143), Bastian et al. (144), Zhou and Xia (145), Szklarczyk et al. (146)
Phylogenetic analysis	Strain tracking	MEGA, PhyML, BEAST	Evolutionary relationships	Model assumptions; sequence quality sensitivity	https://www.megasoftware.net/	Enav et al. (147)

## 8 Challenges and future perspectives

While the *in silico* revolution has provided transformative tools for parasitology, its complete potential is limited by several significant challenges. Tackling these constraints will be essential for the upcoming decade of research.

### 8.1 Validation and data quality

The main obstacle faced by any *in silico* prediction is the requirement for experimental validation (55). A high-scoring virtual hit obtained from a molecular docking screen or a promising epitope identified through an immunoinformatics pipeline must still undergo confirmation *in vitro* and *in vivo*. The effectiveness of computational models in making predictions is directly influenced by the quality and quantity of the training data available. In the field of parasitology, data tends to be sparse, heterogeneous, and frequently sourced from various experimental conditions, which can result in potential biases and a decrease in the generalizability of the (53). Future initiatives should concentrate on the development of standardized data reporting protocols and the establishment of high quality, curated public databases that are specifically tailored to parasitic diseases.

### 8.2 Integration and interoperability

The present state of *in silico* tools is characterized by fragmentation, featuring a multitude of specialized software packages and web servers, each designed to tackle a specific phase in the research pipeline (for instance, gene prediction, docking, epitope mapping) (9). A considerable challenge is posed by the need to integrate these varied tools into cohesive, automated workflows capable of managing the vast scale of contemporary omics data. The creation of user-friendly, integrated platforms that enable researchers to transition smoothly from genomic data to a validated drug or vaccine candidate will be a primary area of focus (55).

### 8.3 The future: personalized and precision parasitology

Looking ahead, the primary objective of *in silico* parasitology is to facilitate precision medicine (100). This entails the application of computational tools to customize interventions according to the specific parasite strain affecting a patient and the unique genetic background of the individual host. For instance, analyzing the genome of a patient’s parasite isolate and employing machine learning models to forecast its drug resistance profile and virulence factors could empower clinicians to promptly choose the most effective treatment (100). Likewise, the integration of host immunogenetic information (such as HLA type) with immunoinformatics may pave the way for the development of personalized vaccines or immunotherapies that are optimally effective for a particular individual (55). The intersection of artificial intelligence, structural biology, and systems modeling heralds a future in which parasitic diseases are addressed not through broad-spectrum, one-size-fits-all methods, but through highly targeted, computationally informed strategies.

## 9 Conclusion

*In silico* techniques have fundamentally transformed the field of parasitology, shifting it from a primarily experimental focus to a more data-centric science. Computational tools now encompass every phase of antiparasitic research—decoding intricate parasite genomes, reconstructing metabolic pathways, strategically designing next-generation vaccines, and discovering drug candidates through virtual screening and structural modeling. The fruitful collaboration between bioinformatics, artificial intelligence, structural biology, and conventional wet-laboratory research has established a robust innovation engine that accelerates discovery and provides renewed optimism in the fight against neglected parasitic diseases.

The sections of this review collectively demonstrate that each computational area—genomics, immunoinformatics, drug discovery, systems biology, AI diagnostics—has its own unique limitations, which vary in their sources but converge on a shared necessity: a close, iterative integration of computational and experimental sciences. As analytical tools advance, datasets become more comprehensive, and interdisciplinary cooperation intensifies, *in silico* parasitology will increasingly lead—rather than simply assist—the global efforts to combat these enduring and evolutionarily adaptable pathogens.
